# Information-seeking in first visit pregnant women in Khayelitsha, South Africa

**DOI:** 10.4102/hsag.v25i0.1478

**Published:** 2020-10-28

**Authors:** Thabani M. Noncungu, Jennifer A. Chipps

**Affiliations:** 1 School of Nursing, Faculty of Community Health Sciences, University of the Western Cape, Cape Town, South Africa

**Keywords:** maternal health, health education, antenatal clinic, low-income setting, information-seeking behaviour, pregnant women, antenatal care, maternal health literacy

## Abstract

**Background:**

The quality of the decisions made by women during pregnancy, especially their first visit, depends on their health needs, their health-seeking behaviour and the type of information available to them.

**Aim:**

This study describes the health education needs, information barriers and health information-seeking behaviour of pregnant women on their first visit to antenatal clinics in a low-income setting in the Western Cape.

**Setting:**

The setting was two antenatal facilities in Khayelitsha Health District facilities in South Africa.

**Methods:**

A quantitative descriptive survey was conducted. A systematic random sample of 261 antenatal first visit attendees between May and July 2016 was selected. Data were collected using a researcher-administered questionnaire and was analysed using descriptive statistics, 95% confidence intervals and non-parametric tests.

**Results:**

The response rate of the study was 92% (*n* = 240). Pregnant women attending an antenatal clinic for the first time reported high information needs with low health information-seeking behaviours and high information barriers. Doctors, nurses (2.2, ±1.0), family and friends (2.0, ±0.6) were the most frequently used sources of health information, while watching television or listening to the radio (1.5, ±0.9) were the least used sources of health information. Having a medical diagnosis (*p* < 0.001) and being of an advanced maternal age (*p* = 0.005) were predictive of higher health-seeking behaviour. The reliance on passively receiving information from health sources may indicate low levels of health literacy and its inverse relationship to health promoting behaviours which should be the subject of further investigation.

## Introduction

In most developing countries, complications during pregnancy and labour are the leading causes of death amongst women of reproductive age (Sialubanje et al. 2014). To ensure good health of pregnant women (Bjelke et al. 2016), antenatal visits, where health risks are identified and managed and health education is provided, play an important role (Al-Ateeq & Al-Rusaiess 2015). Therefore, health education during antenatal visits is an important element of antenatal care (Al-Ateeq & Al-Rusaiess 2015), especially during the first visit to an antenatal care clinic (Maher, Spurling & Askew 2014).

Pregnancy is a time when women might be actively seeking information to protect their growing foetus (Edvardsson et al. 2011; Olander, Smith & Darwin 2018; Willcox et al. 2015). Access to information and maternal health-seeking behaviour is linked to a gap in knowledge, which an individual tries to bridge (Onuoha & Amuda 2013), and is key to obtaining health information during pregnancy (Mulauzi & Daka 2018). Although pregnant women may have a number of information sources to choose from when seeking information regarding pregnancy (Grimes, Forster & Newton 2014), such as the Internet, popular media or family and friends, health workers remain the most frequently used source of information (Ebijuwa, Ogunmodede & Oyetola 2013; McArdle et al. 2015; Owusu-Addo, Owusu-Addo & Morhe 2016; Song et al. 2013). However, access to information is not equitable in low- and high-income settings where differing levels of opportunities and availability of information sources are experienced. In addition, information-seeking is influenced by information needs, information sources, and demographic and clinical pregnancy-related factors, which can enhance information-seeking but may also be barriers (Bernhardt & Felter 2004).

The aim of this study was to investigate the health education needs, barriers to health information and information-seeking behaviour of pregnant women attending an antenatal clinic for the first time at the Khayelitsha Health District facilities in South Africa. This study used Wilson’s model of information-seeking behaviour which proposes that information-seeking is influenced by the relevant information needs and intervening variables, such as information sources and demographics (Bernhardt & Felter 2004).

## Materials and methods

A quantitative, descriptive survey was conducted at two antenatal healthcare facilities in the Khayelitsha Health District in South Africa.

### Setting and population

The Khayelitsha health district is primarily a low-income health district in the Western Cape province of South Africa, with 47.3% of its population having not completed secondary schooling and 46.3% living in informal dwellings. The two purposefully selected antenatal care facilities were situated in large sub-locations, Site B and Harare, which have a high patient intake.

### Study population and sampling strategy

The study population included pregnant women > 18 years of age during their first visit to the two antenatal clinics in Khayelitsha Health District. The sample size was calculated using the following parameters: confidence interval (CI) of 95%, 5% error and 5% uncertainty, resulting in a sample size of 261 first-time attendees. A systematic random sampling strategy was used to ensure the accuracy of obtaining the sample size by approaching every fourth individual who attended the clinic on the selected days during 2 months of data collection.

### Data collection

A researcher-administered questionnaire was used with permission from Shieh, McDaniel and Ke (2009). The questionnaire included demographics and three sections, namely, Section 1: The Pregnancy Health Information Needs Scale (PHINS) which has 20 items on lifestyle, psychological, medication and pregnancy-related needs of pregnant women measured using a five-point Likert scale from strongly disagree (1) to strongly agree (5); Section 2: The Pregnancy Health Information Barrier Scale (PHIBS) which has 15 items on information barriers using a five-point Likert scale from strongly disagree (1) to strongly agree (5); and Section 3: The Pregnancy Health Information-Seeking Scale (PHISS) which has seven information sources and use of these sources was measured using a four-point Likert scale from never (1) to almost all the time (4). The scales had established reliability and validity in an international setting with Cronbach’s alphas of 0.68–0.75 for PHISS, 0.93 for the PHINS, 0.69 for the PHIBS and overall scale < 0.70 (Shieh et al. 2009). The original scales were in English and subsequently were translated and back translated into isiXhosa, the local language used by the clinical attendees. To ensure reliability, the questionnaire was pretested to ensure that it maintained its original meaning. A change was made in the PHIBS where the ‘Health Fairs’ were removed from the scale and the pre-test data were excluded from the study. To test for internal consistency, Cronbach’s alphas were calculated for each scale, with reported Cronbach’s alphas of 0.73 for the PHISS, 0.79 for the PHINS, 0.22 for the PHIBS and 0.64 for the overall scale.

Data collection was conducted for the period of 11 weeks between May and July 2016 using a researcher-administered questionnaire. The data were collected by the researcher and two research assistant (trained in the data collection process and questionnaire) and three nurses who aided with recruitment of women into the study. The administering of each research questionnaire took about 40 min and was administered after the history taking in the morning and before their physical assessment by the midwives.

### Data analysis

The data were captured on the questionnaire and entered, cleaned and analysed using the Statistical Program for Social Science (SPSS) version 21. Descriptive statistic techniques were used to present frequencies and central tendencies (means and standard deviation [SD]) with 95% CIs and Mann–Whitney tests were used to examine the associations between the scales and the demographic variables. A multiple linear regression model was used to assess the ability of the two measures (health information needs [PHINS] and health information barriers [PHIBS]) to predict health information-seeking behaviours (PHIS), after controlling for the influence of significant variables correlated with information-seeking. Preliminary analysis was conducted to ensure no violation of the assumptions of normality, linearity, multicollinearity and homoscedasticity.

### Ethical considerations

Ethical approval of the study was granted by Senate Research Committee of the University of the Western Cape (REC registration number: 15/7/250).

Managers and respondents from the antenatal healthcare facilities provided their written permission to conduct the study. Information about the study was provided verbally to the participants along with a printed information brochure for further reading. Participation in the study was voluntary and data were collected anonymously and treated confidentially. The privacy of data was maintained by placing completed questionnaires in a sealed envelope, stored for 5 years and the electronic data were protected with a password.

## Results

### Demographic and clinical characteristics

A total of 240 respondents (92%) completed the questionnaire. The respondents were aged between 18 and 43 years (mean age: 27 years, SD = 6.0), with two-thirds (63.3%) of the respondents being older than 35 years. Nearly a quarter of the respondents reported their marital status as single (70%), three-quarters (75.8%) of the respondents had reached secondary school level and nearly half (43.3%) of the respondents were unemployed (Table 1).

**TABLE 1:** Demographic characteristics of respondents.

Demographic variables	Number	
	*n*	%
**Maternal age (years)**		
< 35	52	63.3
> 35	88	36.7
**Marital status**		
Single	168	70.0
Married	68	28.3
Divorced/separated	4	1.7
Live with baby’s father	120	50.0
**Educational status**		
Secondary school	182	75.8
University/College	55	22.9
Other	3	1.3
**Occupational status**		
Unemployed	104	43.3
Employed	96	40.0
Student	40	16.7
**Number of pregnancies**		
First pregnancy	94	39.2
2–7 pregnancies	139	60.8
**Gestation age on first visit**		
1–12 weeks	38	15.8
13–27 weeks	72	30.0
28–40 weeks	12	5.0
Average duration of pregnancy	17.2 weeks (± = 6.3)	
Reported medical conditions at first visit	25	10.0

Nearly two-thirds (60.8%) of the respondents reported having had more than one pregnancy with the average gestation age at the time of their first visit being 17.2 (± = 6.3) weeks. Ten per cent (25) of the respondents reported having an additional medical condition (Table 1).

### Health information needs

The PHINS had good reliability with a Cronbach’s alpha of 0.79. The respondents had high overall ratings for the specific health information needs during pregnancy (80.45 [±10.79], [95% CI: 79.17–81.91]) (Table 2). There were significant differences in the ratings of different health information needs with *Information on how the baby grows and develops* (4.56 [±0.81], 4.46–4.66) and *the danger signs of pregnancy* (4.55 [±0.68], [95% CI: 4.47–4.64]) rated significantly higher. Health information on *the proper use of seat belts during pregnancy* (3.17 [±1.51], [95% CI: 2.98–3.36]), *how much weight they should gain* (3.45 [±1.41], [95% CI: 3.27–3.63]) and *HIV test and prevention* (3.49 [±1.62], [95% CI: 3.28–36]) were rated significantly lower (Table 2).

**TABLE 2:** Health information needs measuring pregnancy-related information needs.

Health information needs	Mean (SD)		95% CI
	*n*	%	
How the baby grows and develops	4.56	0.81	4.46–4.66
What are the danger signs of pregnancy	4.55	0.68	4.47–4.64
Information about prenatal vitamins	4.22	1.05	4.09–4.35
Using illegal drugs	4.2	1.12	4.05–4.34
What I should or should not eat	4.2	1.08	4.06–4.33
Deal with stress during pregnancy	4.19	1.22	4.03–4.34
Physical abuse to women by partners	4.18	1.07	4.04–4.31
How to balance rest and activity	4.15	1.24	3.99–4.30
Emotional changes during pregnancy	4.13	1.02	4.00–4.25
Practice safe sex during pregnancy	4.12	1.20	3.97–4.27
Birth control methods to use	4.07	1.28	3.90–4.23
What are safe exercises for me	4.03	1.13	3.88–4.17
Prepare for breast feeding	3.99	1.26	3.83–4.15
Smoking and pregnancy	3.98	1.22	3.82–4.13
Alcohol use and pregnancy	3.97	1.29	3.81–4.13
Kinds of safe and unsafe medications	3.97	1.37	3.79–4.14
What to do if my labour starts early	3.95	1.37	3.78–4.12
HIV test and prevention	3.49	1.62	3.28–3.69
How much weight should I gain	3.45	1.41	3.27–3.63
Proper use seat belts during pregnancy	3.17	1.51	2.98–3.36

**Total PHINS score**	**80.54**	**10.79**	**79.17–81.91**

CI, confidence interval; HIV, human immunodeficiency virus; PHINS, Pregnancy Health Information Needs Scale; SD, standard deviation.

### Health information barriers

Respondents reported high health information barriers (42.27 [±6.57], [95% CI: 41.43–43.10]) during pregnancy (Table 3). *Not having many health activities near home* (3.88 [±1.47], [95% CI: 3.69–4.07]), *no need for information as I already know how to take care of myself* (3.70 [±1.54], [95% CI: 3.51–3.90]), *not much information on the media* (3.37 [±1.36], [95% CI: 3.19–3.54]), *books and magazines are expensive* (3.32 [±1.16], [95% CI: 3.17–3.47]) and not *knowing how to find information* (3.28 [±1.640], [95% CI: 3.07–3.49]) were rated significantly higher than other barriers. *Knowing more information will not help them make medical decisions* was rated significantly lower than the other barriers (2.00 [±1.29], 95% CI: 1.84–2.17) (Table 3). The PHIBS had very poor internal consistency in this population.

**TABLE 3:** Health information barriers measuring pregnancy health information barriers.

Health information barriers	Mean (SD)		95% CI
	*n*	%	
Not many health activities are near my home	3.88	1.47	3.69–4.07
No need for information, already know how to take care of self	3.7	1.54	3.51–3.90
Not much health information on media	3.37	1.36	3.19–3.54
Books and magazines are expensive	3.32	1.16	3.17–3.47
Do not know how to find pregnancy health information	3.28	1.64	3.07–3.49
Not using the computer to learn about pregnancy and health	3.1	1.67	2.89–3.31
Information from healthcare providers is not helpful	2.79	1.46	2.60–2.97
Books or magazines hard to read	2.55	1.34	2.38–2.72
Do not know how to use the internet	2.50	1.60	2.32–2.72
Too much information stresses me out	2.47	1.63	2.26–2.68
Finding a bus or car to library, childbirth classes, hospital is not easy	2.46	1.73	2.24–2.68
Uncomfortable asking doctor or nurse questions	2.38	1.68	2.17–2.60
No friends or family members to answer questions	2.28	1.57	2.07–2.48
Time consuming to find health information	2.17	1.4	1.99–2.34
Knowing more information will not help made medical decisions	2.00	1.29	1.84–2.17

**Total PHIBS score**	**42.27**	**6.57**	**41.43–43.10**

CI, confidence interval; PHIBS, Pregnancy Health Information Barrier Scale; SD, standard deviation.

### Health information-seeking

The PHISS had good internal consistency with a Cronbach’s alpha of 0.73. The respondents reported low use of information sources (4.30 [±3.85], [95% CI: 0.36–0.58]), indicating low health-seeking behaviours for health information (Table 4). Overall, *asking doctor, nurse or other professionals* (1.32 [±1.13], [95% CI: 1.18–1.460]) and *talks given by clinics and hospitals* (0.85 [±1.16], [95% CI: 0.70–1.0]) were scored significantly higher than all other sources (Table 4).

**TABLE 4:** Health information-seeking sources.

Health information sources	Mean (SD)		95% CI
	*n*	%	
Doctor, nurse or other professionals	1.32	1.13	1.18–1.46
Talks given by clinics and hospitals	0.85	1.16	0.70–1.00
Family and friends	0.62	0.67	0.54–0.71
Read books and brochures	0.47	0.88	0.36–0.58
Watch television or listen to radio	0.4	0.75	0.36–0.58
Read newspapers or magazines	0.33	0.71	0.36–0.58
Community health activities	0.32	0.74	0.36–0.58

**Total PHISS score**	**4.3**	**3.85**	**0.36–0.58**

CI, confidence interval; PHISS, Pregnancy Health Information-Seeking Scale; SD, standard deviation.

## Prediction of health information-seeking behaviours

A multiple linear regression model was used to assess the ability of two measures (health information needs [PHINS] and health information barriers [PHIBS]) to PHIS, after controlling for the influence of two significant variables (age and medical diagnosis), which were found to be statistically correlated with information-seeking. The multiple linear regression model was significant (*F* = 6.8, *df* = 4, *p* < 0.044). The positive predictive variables were having a medical diagnosis (6.9 vs. 4.0, *β* = 0.257, *p* < 0.001) and being of normal reproductive age compared to advanced maternal age (4.8 vs. 3.5, *β* = 0.176, *p* = 0.005).

## Discussion

To the best of our knowledge, this study is the first of its kind to provide a snapshot regarding the health-seeking behaviours of pregnant women attending an antenatal clinic for the first time in a low-income setting such as the Khayelitsha Health District facilities in South Africa and builds on the existing of literature on health-seeking behaviours of pregnant women.

The respondents had high information needs similar, but higher, to a previous study in a high-income country, namely, the United States of America (72.66, ±14.56) (Shieh et al. 2009). This difference could be because of the differences in economic status in these two settings which may have a direct influence on the resources and information women perceived to need during pregnancy. The highest rated information needs were around the development of the baby, which was is in line with a study in Sweden that indicated the majority of respondents reported the development of an embryo as being an important information need (Bjelke et al. 2016; Mousavi Chalac & Riahi 2017).

First visit attendees in this setting, however, had very low information-seeking behaviours (4.30, ±3.85). This is in contrast with Shieh et al. (2009) in the United States of America, who reported high information-seeking behaviours (17.56, ±3.78) of pregnant women on their first antenatal visit (Shieh et al. 2009). In this study, two factors explained higher information-seeking behaviours, namely, having a medical diagnosis which explained 26% of the variations in health-seeking behaviour, although only 10% of the group had a medical condition. Similarly, advanced maternal age explained 18% of the variations in health-seeking behaviour, with 38% of the group being over 35 years of age. The influence of maternal age on health-seeking behaviour was supported by a study in Malaysia which found significant positive effects of maternal age on the health-seeking behaviours of respondents (Sutan, Hassan & Shamsuddin 2016).

Low health information-seeking could be influenced by the high information barriers that women experienced in this study in contrast with Shieh et al. (2009) who reported low information barriers to pregnancy-related information in a high-income country. Although the scale had low internal consistency in this setting, the highest rated barrier reported was not having any health activities near them and the lack of information in the media and books. These findings were supported by a study in Nigeria (= 84), also a low-income setting, which reported their respondents having challenges in accessing pregnancy-related information and not having information centres to seek information from (Anasi & Allison 2018).

The low health information-seeking could also be explained by the reliance of respondents on health professionals and clinic talks for health information on pregnancy. This is a universal phenomenon with findings of studies conducted in Saudi Arabia (*n* = 258), the United Kingdom (*n* = 314) and the United States of America (*n* = 70) all reporting that doctors and nurses were a highly regarded source of pregnancy-related information (Clarke, Paterson & Sirota 2019; Ramisetty-Mikler et al. 2018; Zimmerman 2018). In the study setting, health professionals are the most readily available source of information, but this may also indicate a health education culture of passive reception of information rather than active seeking out of information.

The respondents also indicated that asking family and friends for pregnancy-related information 0.85 (±1.16, 0.70–1.00), although this was rated significantly lower than information from health professionals and talks at clinics. Family and friends have been identified as sources of information in other international studies in Iran (*n* = 400) and Rawalpindi (*n* = 208) where nearly three-quarters (72%) and over half (52%) of the respondents sought out information from friends and family (Kamali et al. 2018; Khan & Shahid 2019). In contrast with other studies that identified listening to the radio and watching television as the preferred source of information for pregnant women (Anasi & Allison 2018; Kumara et al. 2019; Obasola & Mabawonku 2018), watching television and listening to the radio were rated significantly lower in this study. This could be because of financial reasons, educational levels, information not been offered on these sources or a lack of trust shown by pregnant women (*n* = 70) in the information provided by broadcast media such as television (Zimmerman 2018).

## Conclusion

Pregnant women attending an antenatal clinic for the first time reported high information needs with low health information-seeking behaviours and high information barriers. Their reliance on passively receiving information from health sources may indicate low levels of health literacy and its inverse relationship to health promoting behaviours; this should be the subject of further investigation.

## Recommendation for practice and education

Health professionals continue to play an important role as the main source of information highlighting the important role in promoting positive outcomes through health education. This study may offer guidance to antenatal nurses working at primary healthcare facilities in identifying the key health information needs of pregnant women. However, the high information needs combined with low health-seeking behaviours and high information barriers highlight the need for health professionals to move away from traditional health education models and move towards health literacy as an outcome of health education based on the needs of pregnant women.

## Limitations

The target population of the study was limited to pregnant women attending an antenatal clinic for the first time, thus excluding the health seeking behaviours of other pregnant attenders. The data sample of the study was derived from two clinics in Khayelitsha. This limits the generalisation of the study results to pregnant women attending the specific antenatal clinics. There could be potential recall bias on health information sources used in the past month. The questionnaire was developed in a high income setting and the low internal consistency of the PHIBS scale suggest that further testing of the questionnaire is recommended.
